# Association of Chronic Periodontitis with *Helicobacter pylori* Infection in Stomach or Mouth: A Systematic Review and Meta-Analysis

**DOI:** 10.1055/s-0042-1756690

**Published:** 2022-11-18

**Authors:** Athanasios Tsimpiris, Ioannis Tsolianos, Andreas Grigoriadis, Ioannis Moschos, Dimitrios G. Goulis, Georgios Kouklakis

**Affiliations:** 1 Department of Medicine, Democritus University of Thrace, Alexandroupolis, Greece; 2 Dental Sector, 424 General Military Training Hospital, Thessaloniki, Greece; 3 Dental School, Faculty of Health Sciences, Aristotle University of Thessaloniki, Thessaloniki, Greece; 4 Department of Preventive Dentistry, Periodontology and Implant Biology, Dental School, Faculty of Health Sciences, Aristotle University of Thessaloniki, Thessaloniki, Greece; 5 Department of Nursing, International Hellenic University, Thessaloniki, Greece; 6 1st Department of Obstetrics and Gynecology, Unit of Reproductive Endocrinology, Medical School, Aristotle University of Thessaloniki, Thessaloniki, Greece; 7 A΄ Department of Pathology, Department of Medicine, Democritus University of Thrace, Alexandroupolis, Greece

**Keywords:** chronic periodontitis, *Helicobacter pylori*, subgingival plaque, stomach, meta-analysis

## Abstract

*Helicobacter pylori* ( *H. pylori* ) infection and periodontitis are both inflammatory conditions associated with systemic diseases. Researchers have attempted to investigate the correlation between them. This systematic review and meta-analyses were conducted to investigate the association of *H. pylori* infection in the stomach and/or in subgingival plaque and gingival crevicular fluid with chronic periodontitis. The protocol was created according to the Preferred Reporting Items for Systematic review and Meta-Analysis Protocols (PRISMA-P) statement. The study was designed according to the Cochrane criteria. A comprehensive literature search was performed in MEDLINE, Scopus, and CENTRAL, combined with hand-searching and assessment of gray literature. The meta-analysis of the included studies was made by the Review Manager (RevMan) 5.4 software. The effect measure of the outcome was odds ratios with 95% confidence intervals. Heterogeneity was assessed by chi-square and I ^2^ . Four observational studies involving 818 subjects were included in this meta-analysis. The odds of oral *H. pylori* presence were higher in patients with chronic periodontitis, compared to healthy controls, with an odds ratio of 1.87 (95% confidence interval 0.85–4.10; p = 0.12). The odds of the presence of *H. pylori* in the stomach also were higher in patients with chronic periodontitis, with an odds ratio of 1.80 (95% confidence interval 0.82–3.95; *p* = 0.15). There is no evidence for an association between chronic periodontitis and the prevalence of *H. pylori* , detected either in subgingival plaque and gingival crevicular fluid or in the stomach.

## Introduction

*Helicobacter pylori* (H. *pylori* ) is a gram-negative, spiral (S-shaped), microaerophilic organism that colonizes the gastric mucosa. 1 It is the leading cause of gastritis, peptic ulcer and gastric cancer, 1 2 mainly transmitted through the oral–oral 3 or fecal–oral routes. 3 4 Although the global prevalence of *H. pylori* infection is more than 50%, 5 6 higher rates are observed in developing countries (51%) compared with developed ones (35%). 7 *H. pylori* infection has been associated with several systemic diseases, such as iron deficiency anemia, 8 cardiovascular disease, 9 10 11 12 type 2 diabetes, 13 14 and pregnancy complications. 15 The diagnosis of *H. pylori* infection is set by the urea breath test (UBT), stool antigen test (SAT), serology, endoscopy, rapid urease test (RUT), histology, and polymerase chain reaction (PCR). Each of these methods carries advantages and disadvantages. 16

Periodontitis is an inflammatory disease of the supporting dental tissues whose manifestation and development are determined by the nature of the immune response to bacterial biofilms. The latter are typically composed of gram-negative microorganisms adhering to the hard dental surfaces, known as dental plaque. 17 18 19 In the advanced form of the disease, destruction of the alveolar bone is caused, which leads to the formation of periodontal pockets and retraction of the gums. 17 20 The prevalence of periodontitis is high, ranging from 20 to 50% worldwide. 21 Periodontal disease has been associated with a variety of chronic diseases, such as cardiovascular disease, 20 21 diabetes, 22 and pregnancy complications. 23

Research efforts focus on understanding the mechanisms of periodontal diseases. Traditional detection methods are insufficient in detecting nonculturable microbial species. On the contrary, metagenomic technology, as it is not based on microbial cultivation but on analysis of the functional genes of the microbial communities, interprets the microbial diversity, the synthesis of metabolic pathways, and the interaction between microorganisms and the environment. 24 25 Metagenomics studies microbial genetic material directly from environmental samples by sequence analysis. 26 This approach might lead to the detection of new and specific periopathogenic bacterial species and clarify the differences between symbiotic and dysbiotic biofilm. The latter is important for understanding the molecular mechanisms of the onset and progression of periodontitis and for providing targeted treatment. 25

The common features of *H. pylori* infection and periodontitis (inflammatory response, association with chronic diseases), 27 as well as the transmission of *H. pylori* through the oral route, led the researchers to investigate colonies in areas within the oral cavity in patients with chronic periodontitis. At the same time, an association has been established between periodontitis and *H. pylori* infection, suggesting that the oral cavity is a potential reservoir of *H. pylori* . 28 29 In interventional studies, successful eradication of gastric *H. pylori* resulted in improved periodontal disease. 30 31

As the studies published so far have been focused on the supragingival plaque or patients with periodontal diseases in general (including gingivitis), the present systematic review and meta-analysis aimed to investigate the association of *H. pylori* infection in the stomach and/or in specific oral cavity areas (subgingival plaque, and gingival crevicular fluid) with chronic periodontitis.

## Methods

### Protocol and Registration

The protocol was created according to the Preferred Reporting Items for Systematic review and Meta-Analysis Protocols (PRISMA-P) statement and registered to the International prospective register of systematic reviews (PROSPERO) database (Record ID: CRD 42021229036).

### Data Sources

A comprehensive search was performed in three electronic databases (MEDLINE/PubMed, Scopus, Cochrane Controlled register of Trials) from conception until January 1st, 2021. Manual searching was performed on Google and Google Scholar. Gray literature was assessed via opengrey.eu, applying the search terms “chronic periodontitis” and “ *H. pylori* .” The search strategy in MEDLINE is presented in Table 1 . 32

**Table 1:** Search strategy in MEDLINE

Search	Query
#1	((((((((((((generalized periodontitis) OR (chronic periodontal inflammation)) OR (periodontitis)) OR (chronic periodontitis)) OR (mild periodontal disease)) OR (moderate periodontal disease)) OR (advanced periodontal disease)) OR (severe periodontal disease)) OR (periodontal disease)) OR (CP)) OR (periodontal disease[MeSH Terms])) OR (periodontitis[MeSH Terms])) OR (chronic periodontitis[MeSH Terms])
#2	(((((Helicobacter pylori) OR (H. pylori)) OR (H pylori)) OR (Campylobacter pylori)) OR (helicobacter pylori[MeSH Terms])) AND (((((((((((((((deep periodontal lesion) OR (pocket with deep probing depth*)) OR (site with deep probing depth*)) OR (pocket with probing depth* >5mm)) OR (pocket with probing depth* 6mm)) OR (site with probing depth* >5mm)) OR (site with probing depth* 6mm)) OR (dental plaque)) OR (subgingival plaque)) OR (periodontal pocket)) OR (gingival crevicular fluid)) OR (GCF)) OR (dental plaque[MeSH Terms])) OR (periodontal pocket[MeSH Terms])) OR (gingival crevicular fluid[MeSH Terms]))
#3	(((((Helicobacter pylori) OR (H Pylori)) OR (H. Pylori)) OR (Campylobacter pylori)) OR (helicobacter pylori[MeSH Terms])) AND (((((((((((stomach) OR (gastric)) OR (gastric mucosa)) OR (stomach antrum)) OR (pylorus)) OR (gastric epithelium)) OR (pyloric antrum)) OR (stomach[MeSH Terms])) OR (gastric mucosa[MeSH Terms])) OR (pyloric antrum[MeSH Terms])) OR (pylorus[MeSH Terms]))
#4	#2 OR #3
#5	#1 AND #4

### Inclusion and Exclusion Criteria

The studies were considered eligible if they (i) were randomized controlled trials and of observational type (cohort, cross-sectional, case-control) studies, (ii) were approved by ethics committees, (iii) were written in English, (iv) reported relevant data on two study arms [(a) patients with chronic periodontitis, (b) healthy controls], and (v) had adopted specific criteria for the definition of chronic periodontitis.

The diagnosis of chronic periodontitis had to be based on clinical or/and radiographic criteria, according to the 1999 classification system 33 or the 1989 classification system. 34 The studies were excluded if they (i) were of a low level of evidence (case-reports, case-series), (ii) included non-adult populations, and (iii) referred to specific conditions, namely pregnancy, orthodontic treatment, systemic diseases, malignancies, diabetes mellitus, auto-immune diseases, chronic use of non-steroidal anti-inflammatory drugs, antibiotics, proton pump inhibitors and bismuth salts use during the last two months, periodontal treatment (scaling, root planning) during the last six months, history of *H. pylori* eradication, gastrectomy, and less than 20 remaining teeth.

### Study Records

Citations exported by the electronic databases in compatible file versions were imported to the Mendeley platform for managing study records. After removing the duplications, the records were exported to the Rayyan platform. 35 After reading the title and abstract, two reviewers (AG, IT) decided independently about the study eligibility. In relevant studies, the full text was assessed by two reviewers (AG, IT) independently. Conflicts were solved by a third reviewer (AT).

### Data Extraction

A Microsoft Excel sheet was used for data extraction. Study identification data (name of the first author, year of publication, country) and population data (age, gender, sample size) were recorded. Regarding chronic periodontitis, the number of cases and controls were recorded. Regarding *H. pylori* infection, the number of positive and negative subjects (among total sample and cases with chronic periodontitis), diagnostic methods (histology, culture, rapid urease test [RUT], urea breath test, enzyme-linked immunosorbent assay, polymerase chain reaction [PCR], stool antigen test), and areas in which *H. pylori* was assessed (stomach, gingival crevicular fluid, subgingival plaque, periodontal pocket) were recorded. Data were extracted by two reviewers (AG, IT) independently. Conflicts were solved by a third reviewer (AT).

### Outcomes

The outcome of the systematic review was the prevalence of *H. pylori* in chronic periodontitis and healthy control arms. The prevalence of *H. pylori* in the stomach and/or in specific oral cavity areas (gingival crevicular fluid, subgingival plaque) was recorded where available.

### Bias Assessment and Confidence

The Newcastle-Ottawa Scale (NOS) was applied to assess the quality of observational studies. 36 Based on the collected quality stars, selection, comparability, and exposure (case-control studies)/outcome (cohort and cross-sectional studies) bias were evaluated as “low”, “high” or “unclear” by two reviewers (AG, IT) independently. Conflicts were solved by a third reviewer (AT).

The Grading of recommendations, assessment, development, and evaluations (GRADE) tool was applied to assess the strength of the evidence. 37 Two reviewers (AG, IT) independently evaluated the evidence of the included studies as “high,” “moderate,” “low,” or “very low.” Conflicts were solved by a third reviewer (AT).

### Statistical Analysis

The meta-analysis of the included studies was made by the Review Manager (RevMan) 5.4 software. The effect measure of the outcome (presence of *H. pylori* —binary) was odds ratios (OR) with 95% confidence intervals (CI). For the quantitative synthesis, a random-effects model (inverse variance) was applied. Heterogeneity was assessed by chi-square and I ^2^ . Subgroup analyses were performed based on the diagnostic method of *H. pylori* and the oral cavity area of *H. pylori* infection.

## Results

**Fig. 1:**
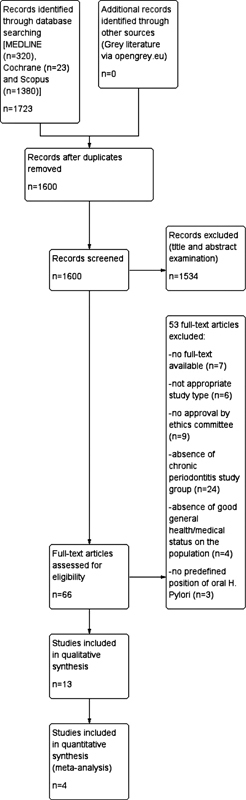
Preferred Reporting Items for Systematic review and Meta-Analysis (PRISMA) flowchart.

The literature search located 1723 studies. After duplicate removal, 1600 studies were assessed based on the title and abstract. Of them, 66 studies were examined as full-text articles, and 13 were included in the qualitative synthesis (PRISMA flowchart— Fig. 1 ). The reasons for exclusion are presented in Table 2 . Four studies 38 39 40 41 were included in the quantitative synthesis (meta-analysis), as nine were excluded for an unclear definition of chronic periodontitis or violated the rule of independent observations in samples.

**Table 2:** List of excluded studies with rationale

Number	Study	Reason for exclusion
1	Al Asqah, 2019	No full-text available
2	Badea, 2002	No full-text available
3	Bielanski, 1999	No full-text available
4	Bussac, 1999	No full-text available
5	Esfahanizadeh, 2010	No full-text available
6	Safarov, 2002	No full-text available
7	Wei, 2020	No full-text available
8	Azzi, 2017	Not appropriate study type
9	Paladino, 2015	Not appropriate study type
10	Payão, 2016	Not appropriate study type
11	Ronellenfitsch, 2016	Not appropriate study type
12	Sujatha et al 2015 58	Not appropriate study type
13	Watts, 2006	Not appropriate study type
14	Al Refai, 2002	No approval by an ethics committee
15	Asikainen et al 1994 46	No approval by an ethics committee
16	Dye et al 2002 42	No approval by an ethics committee
17	Gao, 2011	No approval by an ethics committee
18	Gebara, 2004	No approval by an ethics committee
19	Gebara, 2006	No approval by an ethics committee
20	Riggio and Lennon 1999 44	No approval by an ethics committee
21	YanSong, 2014	No approval by an ethics committee
22	Zheng, 2015	No approval by an ethics committee
23	Adachi, 2019	Absence of chronic periodontitis study group
24	Alagl, 2019	Absence of chronic periodontitis study group
25	Anand et al 2006 56	Absence of chronic periodontitis study group
26	Bago, 2011	Absence of chronic periodontitis study group
27	Berroteran, 2002	Absence of chronic periodontitis study group
28	Bharath, 2014	Absence of chronic periodontitis study group
29	Boylan, 2014	Absence of chronic periodontitis study group
30	Choudhury, 2003	Absence of chronic periodontitis study group
31	Contractor, 1998	Absence of chronic periodontitis study group
32	Czesnikiewicz-Guzik, 2005	Absence of chronic periodontitis study group
33	Ding, 2015	Absence of chronic periodontitis study group
34	Dowsett, 1999	Absence of chronic periodontitis study group
35	Gülseren, 2016	Absence of chronic periodontitis study group
36	Karczewska, 2002	Absence of chronic periodontitis study group
37	Liu, 2009	Absence of chronic periodontitis study group
38	Medina, 2010	Absence of chronic periodontitis study group
39	Namiot, 2006	Absence of chronic periodontitis study group
40	Rajendran, 2009	Absence of chronic periodontitis study group
41	Salazar, 2012	Absence of chronic periodontitis study group
42	Schwahn, 2018	Absence of chronic periodontitis study group
43	Teoman, 2007	Absence of chronic periodontitis study group
44	Tongtawee et al 2019 30	Absence of chronic periodontitis study group
45	Tsami, 2011	Absence of chronic periodontitis study group
46	Zahedi, 2017	Absence of chronic periodontitis study group
47	Bürgers, 2008	Absence of good general health/medical status in the population
48	Flores-Treviño, 2019	Absence of good general health/medical status in the population
49	Hardo et al 1995 47	Absence of good general health/medical status in the population
50	Yang, 2016	Absence of good general health/medical status in the population
51	Bali, 2010	No predefined position of oral *Helicobacter* pylori
52	Suzuki, 2008	No predefined position of oral *H. pylori*
53	Umeda, 2003	No predefined position of oral *H. pylori*

The summary of the characteristics of studies included in the meta-analysis is presented in Table 3 . The characteristics of excluded studies are presented in Tables 4 , 5 , and 6 .

**Table 3:** Summary of studies included in the meta-analysis

Study	First author	Al Asqah et al 38	Nisha et al 39	Salehi et al 40	Silva et al 41
	Year	2009	2016	2013	2010
	Country	Saudi Arabia	India	Iran	Brazil
Population	Sex (M/F)	56/45	239/261	42/58	47/68
	Age (y)	Mean (SD): 40.77 (14.15)	Range, 18–60	Mean (SD): 35.3 (10.6)	Mean (SD): 49.6 (5.8)
	Sample size	101	500	100	115
Chronic periodontitis	Cases	62	293	50	62
	Controls	39	207	50	53
	Definition	Bleeding on probing and at least four teeth with a probing depth ≥3 mm	One or more sites with a probing depth ≥4 mm and clinical attachment loss ≥4 mm at the same site	3 mm clinical attachment loss within at least four teeth and more than 10% of sites with bleeding on probing	At least four different teeth with periodontal pockets ≥5 mm and clinical attachment level >3 mm
Oral *Helicobacter* *pylori*	Positive	66	270	21	0
	Negative	35	230	79	115
	Chronic periodontitis- *H. pylori* positive	49	180	9	0
	Chronic periodontitis- *H. pylori* negative	13	113	41	62
	Detection method	RUT	RUT	PCR	PCR
	Exact location	Subgingival plaque	Subgingival plaque	GCF	Subgingival plaque
*H. pylori* in the stomach	Positive	50	345	N/A	N/A
	Negative	51	155	N/A	N/A
	Chronic periodontitis- *H. pylori* positive	37	209	N/A	N/A
	Chronic periodontitis- *H. pylori* negative	25	84	N/A	N/A
	Detection method	RUT	Serology	N/A	Histology and PCR

Abbreviations: GCF, gingival crevicular fluid; N/A, not available; PCR, polymerase chain reaction; RUT, rapid urease test; SD, standard deviation.

**Table 4:** Summary of demographic characteristics and chronic periodontitis status in studies excluded from the meta-analysis

Sl. No.	Study			Population			Chronic periodontitis		
	First author	Year	Country	Sex (M/F)	Age (y)	Sample size	Cases	Controls	Definition
1	Agarwal	2012	India	28/22	Range: 30–65	50	50	0	N/A
2	Eskandari	2010	Iran	31/36	Mean (SD): 42.3 (12.52)	67	67	0	Periodontal pocket with a depth ≥4 mm and bleeding on probing
3	Gonçalves	2009	Brazil	13/18	≥ 21	31	17	14	At least three sites with probing depth ≥ 5 mm and/or clinical attachment level ≥ 4 mm and bleeding on probing
4	Hu	2016	China	14/0	Range: 18–60	28 samples/14 subjects	14	0	American Academy of Periodontology More than 30% of sites with probing depth deeper than 4 mm, more than 30% of sites with attachment loss of 2 mm
5	Kadota	2020	Japan	13/26	Mean (SD): 35.3(15.1)	39	16	23	Periodontal depth ≥4 mm at third molars
6	Souto	2008	Brazil	N/A	N/A	225	169	56	≥10% of teeth with probing depth and/or clinical attachment loss ≥5 mm, or ≥15% of teeth with the periodontal depth and/or clinical attachment loss ≥4 mm, and >10% of sites with bleeding on probing
7	Tahbaz	2017	Iran	44/56	N/A	100	50	50	N/A
8	Ustaoglu	2018	Turkey	81/74	Range: 18-65	155	60	95	N/A
9	Venkata	2017	India	23/22	Mean: 39	45	30	15	Periodontal depth ≥ 5 mm at more than 30% of sites with relative attachment level ≥ 3 mm and more than 10% of sites with bleeding on probing

Abbreviations: N/A, not available; SD, standard deviation.

**Table 5:** Oral *H. pylori* status in studies excluded from the meta-analysis

Sl. no.	Positive	Negative	Chronic periodontitis— *Helicobacter* *pylori* positive	Chronic periodontitis— *H. pylori* negative	Detection method	Exact location
1	PCR:21/Culture:9	PCR:29/culture: 41	PCR:21/culture: 9	PCR:29/culture: 41	PCR and culture	Subgingival plaque
2	4	63	4	63	PCR	Supra- and subgingival plaque
3	Mean frequency detection (SD): 33 (47)		Mean frequency detection (SD): 50 (33)	Mean frequency detection (SD): 12 (20)	PCR	Subgingival plaque
4	9 a	8 a	9 a	8 a	PCR	Subgingival plaque
5	5 b	18 b	3 b	13 b	PCR	Dental plaque
6	33.3% of subgingival biofilm samples	66.6% of subgingival biofilm samples	50% of samples	50% of samples	PCR	Subgingival plaque
7	5	95	4	96	PCR	Subgingival plaque
8	0	155	0	60	PCR	Subgingival plaque
9	N/A	N/A	N/A	N/A	PCR	Subgingival plaque

Abbreviations: N/A, not available; PCR, polymerase chain reaction; SD, standard deviation.

a The sum of positive and negative cases is not equal to the given sample size

b Number out of extracted third molars.

**Table 6:** *Helicobacter pylori* in the stomach in studies excluded from the meta-analysis

Sl. no.	Positive	Negative	Chronic periodontitis— *Helicobacter pylori* positive	Chronic periodontitis— *H. pylori* negative	Detection method
1	30	20	30	20	Histology and RUT
2	23	44	23	44	RUT
3	N/A	N/A	N/A	N/A	N/A
4	N/A	N/A	N/A	N/A	N/A
5	N/A	N/A	N/A	N/A	N/A
6	N/A	N/A	N/A	N/A	N/A
7	7	93	5	45	N/A
8	N/A	N/A	N/A	N/A	N/A
9	N/A	N/A	N/A	N/A	N/A

Abbreviations: N/A, not available; RUT, rapid urease test.

### Risk of Bias Assessment

**Fig. 2:**
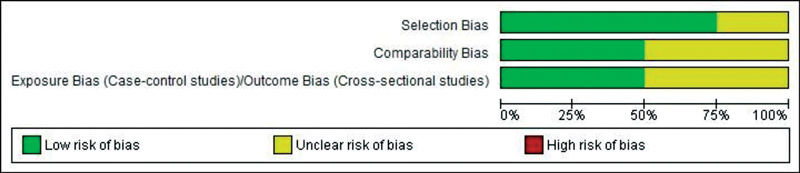
Newcastle-Ottawa Scale. Risk of bias graph: review authors' judgments about each risk of bias item presented as percentages across all included studies.

**Fig. 3:**
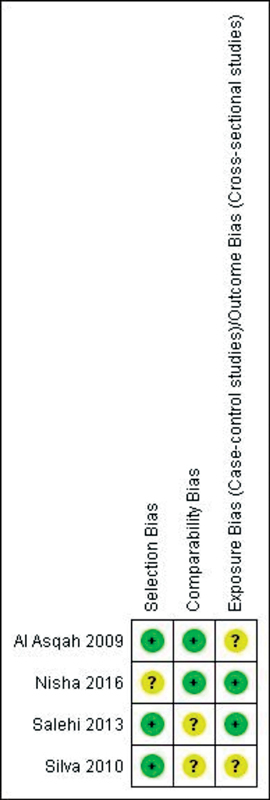
Newcastle-Ottawa Scale. Risk of bias summary: review authors' judgments about each risk of bias item for each included study.

The quality of the included studies was assessed by NOS. According to NOS, the risk of bias was low ( Fig. 2 ). A detailed graph of bias items for each included study is presented in Fig. 3 .

### Association between *H. pylori* and Chronic Periodontitis

**Fig. 4:**
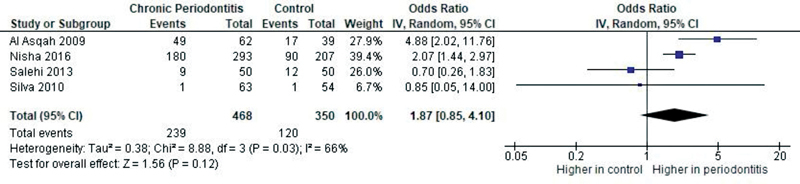
Forest plot of comparison: Presence of *Helicobacter pylori* , outcome: Prevalence of oral *H. pylori.* CI, confidence interval; IV, intravenous.

**Fig. 5:**
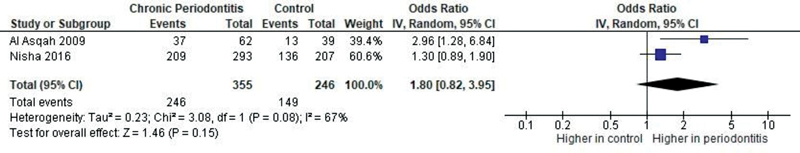
Forest plot of comparison: Presence of *Helicobacter pylori* , outcome: Prevalence of *H. pylori* in the stomach. CI, confidence interval; IV, intravenous.

The odds of presence of oral *H. pylori* in patients with chronic periodontitis were higher compared with healthy controls for oral (OR = 1.87, *p* = 0.12— Fig. 4 ) and stomach (OR = 1.80, *p* = 0.15— Fig. 5 ) detections.

### Subgroup Analyses

**Fig. 6:**
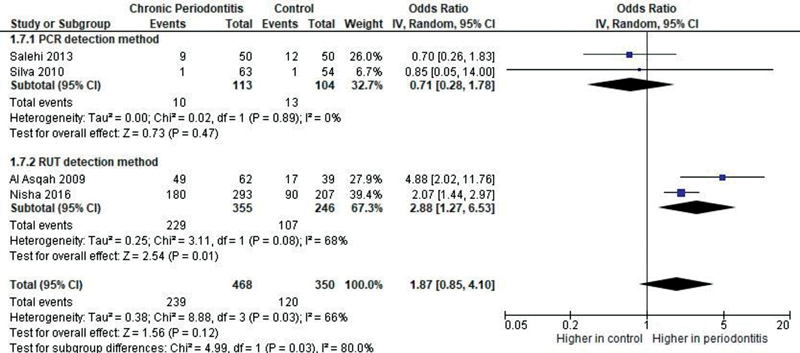
Forest plot of comparison: Presence of *Helicobacter pylori* , outcome: Prevalence of oral *H. pylori* . Subgroup analysis based on detection method. CI, confidence interval; IV, intravenous; PCR, polymerase chain reaction; RUT, rapid urease test.

Subgroup analysis was performed based on the detection method of oral *H. pylori* . When PCR was applied, the odds of the presence of oral *H. pylori* in patients with chronic periodontitis were lower compared with healthy controls (OR = 0.71, *p* = 0.47— Fig. 6 ). When RUT was applied, the odds were higher (OR = 2.88, *p* = 0.01— Fig. 6 ).

### Sensitivity Analyses

**Fig. 7:**
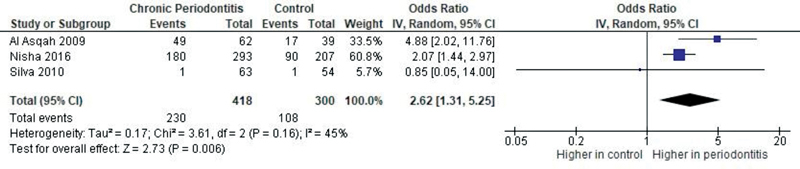
Forest plot of comparison: Presence of *Helicobacter pylori* , outcome: Prevalence of *H. pylori* in subgingival plaque. CI, confidence interval; IV, intravenous.

**Fig. 8:**
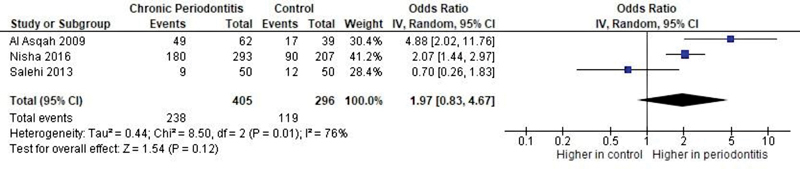
Forest plot of comparison: Presence of *Helicobacter pylori* , outcome: Prevalence of oral *H. pylori* . Sensitivity analysis (study of Silva et al 41 excluded). CI, confidence interval; IV, intravenous.

The study results were not changed after excluding the Salehi et al 40 (reason: *H. pylori* detected in gingival crevicular fluid— Fig. 7 ) and Silva et al 41 studies (reason: zero-count correction— Fig. 8 ).

### Evaluation for Publication Bias

Publication bias could not be assessed as the meta-analysis included only four studies.

### Strength of the Evidence

The GRADE tool was used to assess the strength of the evidence. As all included studies were observational, their initial rating was low. Based on the predefined GRADE criteria, the overall strength of the evidence was low ( Table 7 ).

**Table 7:** GRADE-Strength of the evidence

First author	Al Asqah et al 38	Nisha et al 39	Salehi et al 40	Silva et al 41
Year	2009	2016	2013	2010
Study type	Case–control	Cross-sectional	Case–control	Case–control
Initial rating	Low	Low	Low	Low
Comparison	Patients with chronic periodontitis vs. healthy controls	Patients with chronic periodontitis vs. healthy controls	Patients with chronic periodontitis vs. healthy controls	Patients with chronic periodontitis vs. healthy controls
Outcome—prevalence of *H. pylori*	RUT (oral *Helicobacter pylori* )/ RUT ( *H. pylori* in the stomach)	RUT (oral *H. pylori* )/ Serology ( *H. pylori* in the stomach)	PCR (oral *H. pylori* )/PCR, histology ( *H. pylori* in the stomach)	PCR (oral *H. pylori* )
Study limitations (risk of bias)	Low risk (no reason to downgrade)	Low risk (no reason to downgrade)	Low risk (no reason to downgrade)	Unclear risk (-1)
Inconsistency	Not applicable no reason to downgrade)	Not applicable (no reason to downgrade)	Not applicable (no reasons to downgrade)	Not applicable (no reason to downgrade)
Indirectness of evidence	Direct evidence (no reason to downgrade)	Direct evidence (no reason to downgrade)	Direct evidence (no reason to downgrade)	Direct evidence (no reason to downgrade)
Imprecision	Wide CI (−1)	Not wide CI (no reason to downgrade)	Wide CI (−1)	Not applicable (no reason to upgrade)
Publication bias	Not applicable (no reason to upgrade)	Not applicable (no reason to upgrade)	Not applicable (no reason to upgrade)	Not applicable (no reason to upgrade)
Magnitude of effect	OR > 2. Large effect (+1)	OR > 2. Large effect (+1)	Moderate effect	Not available
Dose–response relationship	Not available data (no reason to upgrade)	Not available data (no reason to upgrade)	Severity of periodontitis affected *H. pylori* , but not statistically significant (+1)	Not available data (no reason to upgrade)
All plausible biases—confounders	No additional confounders referred	Residual confounders referred sufficiently (+1)	No additional confounders referred	No additional confounders referred
Final rating	Low	High	Low	Very low

Abbreviations: CI, confidence interval; OR, odds ratio; PCR, polymerase chain reaction; RUT, rapid urease test.

## Discussion

The role of chronic periodontitis in the recurrence of *H. pylori* infection and/or the resistance to gastric *H. pylori* eradication has been demonstrated by several studies. 42 Α two-way association between these two disease entities has been suggested. 29 The present meta-analysis provided evidence for an association between the presence of *H. pylori* in the subgingival plaque and chronic periodontal disease, as *H. pylori* was detected at a higher rate in the subgingival plaque of patients with periodontitis compared with healthy controls. This finding is consistent with a recent meta-analysis, which concluded that periodontitis is associated with oral *H. pylori* infection due to the presence of the bacterium in saliva and plaque in general. 43 Furthermore, original studies 44 45 using the PCR method arrived at the same conclusion by demonstrating the subgingival plaque as a supply reservoir of *H. pylori* infection in patients with periodontitis. However, other studies did not detect *H. pylori* in the subgingival plaque of patients with chronic periodontitis using the same method. 46 47 48 The reason for this divergence may be the differences in methodological procedures, population samples, 49 50 PCR primers, 51 52 sampling methods, and protocols. 51 Even the collection of the subgingival sample by paper cones differs from the use of periodontal curettes, as the cones can carry a smaller and, therefore, undetectable microbial load. 44 This fact may be the reason why, in the present meta-analysis, the significant association between subgingival *H. pylori* and periodontitis is lost when the sample includes Gingival Crevicular Fluid (GCF).

Another reason for the divergence could be the transient presence of *H. pylori* in the oral cavity. Some authors argue that *H. pylori* exists in the oral cavity only as a transient organism, as other competing species colonize and predominate. 53 *H. pylori* infection may be indirectly related to periodontitis via periopathogenic oral cavity microbes that can compete and bind *H. pylori* strains. This binding of *H. pylori* by periodontal disease bacteria may lead to a cross-antigenicity of *H. pylori* and periopathogens through heat shock proteins, resulting in an increased inflammatory immune response. 53 54 Furthermore, the transient presence of *H. pylori* in the oral cavity may be due to its contamination by gastric fluid that reflux from the stomach. 47 55

The present study concluded that gastric *H. pylori* infection is not associated with periodontal disease, consistent with part 56 57 but not all of the literature. 38 58 Studies have supported the correlation between the *H. pylori* presence in the stomach and periodontitis, concluding that periodontal treatment contributes to the most effective and long-lasting eradication of gastric *H. pylori* . 30 59 However, the possibility of different *H. pylori* genotypes in the oral cavity and stomach of the same individual 60 61 may be the reason for the additional diagnostic difficulty. Cześnikiewicz-Guzik et al 62 did not find an association between the occurrence of *H. pylori* in the stomach and the oral cavity. This finding suggests that other factors, such as susceptibility to infection due to the acidic environment in the stomach, are the main cause of gastric infection with the bacterium. At the same time, the oral cavity can only serve as a means of transient food-related *H. pylori* contamination.

In the present meta-analysis, the correlation between subgingival *H. pylori* and periodontitis was significant only when *H. pylori* was detected by RUT, while this was not the case with PCR. RUT sensitivity ranges from 77 to more than 90%, and its specificity from 98 to 100%. 63 64 65 66 Song et al 60 concluded that the oral cavity may be a permanent *H. pylori* reservoir that can host multiple strains of the bacterium. The different sensitivity of the methods to different *H. pylori* strains could explain why RUT detected a higher percentage of *H. pylori* , as in the PCR method, depending on used primers amplificated specific strains. However, false-positive results of the RUT method are possible under certain conditions, as microorganisms, such as *Klebsiella pneumoniae, Staphylococcus aureus, Proteus mirabilis, Enterobacter cloacae,* and *Citrobacter freundii* , which colonize the oral cavity and/or stomach, have urease activity. 16 On the other hand, one possible reason that PCR detected *H. pylori* more frequently in controls could be the method's main disadvantage, which is the detection of non-living bacteria. 67

Two of this study's strengths are the comprehensive literature search and the assessment of the gray literature to restrict publication bias. Detecting *H. pylori* in both subgingival plaque and gingival crevicular fluid provides a better understanding of the association between the presence of *H. pylori* and chronic periodontitis, given the limited evidence from the literature. One additional strength of this review is the focus on chronic periodontitis, whereas most studies have assessed the presence of *H. pylori* in periodontal diseases in general, including gingivitis.

A couple of limitations are also observed in this study. The number of selected studies was low, restricting authors from conducting additional analyses, such as funnel plots. In each of these studies, a different method for detecting gastric *H. pylori* was performed, which can be explained by the absence of a gold standard detection method. In addition, an alternative of zero-count correction was performed by adding one event in each of the cells of study results by Silva et al. Although, in some meta-analysis tools, this procedure is made automatically by adding 0.5 in each of the cells, no difference was observed in the results by either including or excluding the study mentioned above, leading authors to make this amendment.

Although the term chronic periodontitis has been sufficiently described in previous classification systems, all subjects with periodontal pockets being more than 3 mm were considered periodontitis cases. In addition, it was not feasible to spot any studies in which *H. pylori* was detected in periodontal pockets, as it was designed in the protocol.

Future studies should be more specific regarding the level of periodontal destruction to investigate in detail whether there is a dose–response association between the presence of *H. pylori* and the stages of chronic periodontitis. There is also a need for more studies assessing *H. pylori* in gingival crevicular fluid, as the current evidence is limited.

In summary, there is no evidence of an association between chronic periodontitis and the prevalence of *H. pylori* , when the latter is detected either in specific oral cavity areas or in the stomach. The detection method of oral *H. pylori* can play an important role in affecting this association.
